# Heterogeneity of Cervical Cancer-Associated Tertiary Lymphoid Structures (TLSs) and Their Specific Interrelation With Clinicopathological Parameters

**DOI:** 10.7759/cureus.59077

**Published:** 2024-04-26

**Authors:** Lavinia Bălan, Anca Maria Cimpean, Cristina Secosan, Virgiliu-Bogdan Sorop, Catalin Balan, Mihaela Moldovan, Eugen Melnic, Ligia Balulescu, Simona Brasoveanu, Laurentiu Pirtea

**Affiliations:** 1 Department of Obstetrics and Gynecology, Victor Babes University of Medicine and Pharmacy, Timisoara, ROU; 2 Department of Medicine, Victor Babes University of Medicine and Pharmacy, Timisoara, ROU; 3 Department of Microscopic Morphology/Histology, Victor Babes University of Medicine and Pharmacy, Timisoara, ROU; 4 Center of Expertise for Rare Vascular Disease in Children, Louis Turcanu Children Hospital, Timisoara, ROU; 5 Department of Cell and Molecular Biology, Victor Babes University of Medicine and Pharmacy, Timisoara, ROU; 6 Department of Pathology, Municipal Emergency Hospital, Timisoara, ROU; 7 Department of Pathology, Nicolae Testemitanu State University of Medicine and Pharmacy, Chisinau, MDA

**Keywords:** tertiary lymphoid structure, inflammation, tls.n, prognosis, cervical cancer

## Abstract

Objective: The study investigates morphological variants of tertiary lymphoid structures (TLSs) in relation to cervical cancer development, from intraepithelial neoplastic lesions to invasive carcinomas with locoregional lymph node metastases.

Materials and methods: This retrospective analysis comprised 100 cervical cancer cases who had had total hysterectomy with lymphadenectomy in the Obstetrics and Gynecology Clinic of the Municipal Emergency Clinical Hospital of Timisoara, Romania, from 2020 to 2023. Bilateral ilio obturator lymphadenectomy and total hysterectomy were used to acquire biopsy samples. The presence of germinal centers, other stromal structures, TLS density, topography relative to the tumor lesion, and malignant cell islets are used to evaluate and classify TLS.

Results: We first globally evaluated the total number of TLSs (TLS.T). We observed topographically two places in the cervical stroma: TLS immediately peritumorally positioned and TLS away from tumor lesions. Invasive carcinomas have bigger superficial TLSs with a well-defined germinal center. As they approached the tumor, TLSs increased in size and density. We also detected a special type of TLS associated with nerve fibers, which we named tertiary lymphoid structures associated with nerves (TLS.N). The total number of TLSs did not correlate with age, but 85.71% of patients presenting TLS.N were aged between 59 and 72 years old. Our findings showed a strong correlation between age (postmenopausal, p = 0.005) and TLS-N presence. Similarly, TLS parameters evolved with tumor differentiation. Only in the TLS.N group did the tumoral grading (G) 3 correlate with TLS (p = 0.041), while TLS.T did not correlate with G. All TLS.N. patients, except one, had lymphovascular invasion and massive histiocytosis. On the first point, TLS.N correlated with lymphovascular invasion (p = 0.032).

Conclusion: Tertiary lymphoid structures associated with nerves have not been previously reported in cervical cancer, and their effects on prognosis and aggression are unknown. There was a substantial association between TLSs.N presence and age over 60, suggesting it is exclusive to menopausal women. They were also substantially connected with lymphovascular invasion and G3, suggesting they may be a poor cervical cancer prognostic factor.

## Introduction

Tertiary lymphoid structures (TLSs) are collections of organized lymphoid tissue in the form of structures that mimic classical lymphoid follicles in normal lymph nodes. Tertiary lymphoid structures can be found in normal conditions, but most develop during chronic inflammatory autoimmune lesions or malignant transformations [1-3]. Tertiary lymphoid structures are not a novelty in the autoimmune and tumor-inflammatory fields, but interest in them has greatly increased with the application of anti-programmed cell death protein 1 (PD-1)/programmed cell death ligand 1 (PD-L1) therapies in malignant tumors. Several TLS malignancies have been reported to be prognostic and responsive to major therapy [4]. Considered an immune tissue defense reaction developed in response to malignant transformation, TLSs have been extensively studied in malignant lesions of the breast [5-7], esophageal cancers [8], and malignant melanoma [9]. In all these types of malignant lesions, the existence of TLSs is considered a favorable prognostic factor and an optimal response to therapy [5-9].

There are several malignancies where TLSs are poorly studied and their prognostic and therapeutic impact is incompletely elucidated. Among these are cervical cancers, for which there are only four articles in the literature [10-13]. There is very little data on the timing of TLSs during the course of cervical cancer, and the effect of radiotherapy on their progression and development in the tumor stroma adjacent to malignant epithelial lesions in cervical cancer is not yet known. The TLSs associated with the stroma in the vicinity of the intraepithelial malignant lesions have not been described, nor have their existence and distribution in relation to the anatomical markers of the cervix [11].

The initiation of TLS formation is driven by the increased permeability of small capillary vessels and postcapillary veins in the tumor stroma, followed by the extravasation of lymphocytes into the connective tissue of the cervix with precancerous and cancerous lesions [14]. Despite this, there are no data that the existence of TLSs is influenced by the value of lymphocytes in the peripheral blood of patients with cervical cancer. Cervical malignancies and adjacent lymphoid lesions are often characterized by extensive histiocytosis, but as with peripheral blood lymphocytes, no correlation has been reported to date between TLS presence and peripheral blood monocyte counts. The development of TLS is carried out in a series of successive stages, which explains why a functional morphological and molecular heterogeneity of TLS has been reported in different types of cancer [15]. The morphological heterogeneity is closely correlated with the functional heterogeneity of TLS, which has a different prognosis and therapeutic impact for each type of cancer [15].

The limited information on the presence of TLSs in cervical cancers and the absence of clear data on their prognostic and therapeutic impact have determined the objectives of this study. Due to the fact that TLSs are highly interconnected with the immune milieu in malignant disease, we aim to find if there are any correlations between their presence in the malignant stroma and blood parameters such as lymphocytes, leucocytes, or monocytes in order to include these parameters as potential prognostic markers at the time of diagnosis and be able to anticipate responses to some targeted therapies. The second aim of the study was to describe morphological variants of TLSs in relation to cervical cancer development, from intraepithelial neoplastic lesions to invasive carcinomas with locoregional lymph node metastases, and also to highlight a particular form of TLS that associates a nerve component with well-known components of classic TLSs. These nerve-associated TLS have not been previously described in cervical cancer, and no data about their impact on cervical cancer prognosis or aggressiveness are available at this moment. We found a significant correlation between TLSs associated with nerve (TLS.N) presence and age over 60, suggesting that TLS.N may be specific for menopausal women. Also, they were significantly correlated to lymphovascular invasion and grading (G) 3, and thus, we may assume that their presence may be considered a poor prognostic factor in cervical cancer.

## Materials and methods

The present study is a retrospective study that includes a total of 100 patient cases. Two experienced anatomopathologists selected these cases by re-evaluating microscopically all the paraffin block biopsy samples from 150 cases diagnosed with cervical cancer and undergoing total hysterectomy with bilateral ilio obturator lymphadenectomy during the period selected for the study (2022-2023) in the Obstetrics and Gynecology Clinic of the Municipal Emergency Clinical Hospital of Timisoara, Romania. After applying the exclusion criteria, 100 cases were remaining in the study that met all the criteria. Bioptic samples were processed in accordance with routine pathologic processing rules, and we cut the samples to a size of 1 cm3. Bioptic samples were obtained by total hysterectomy with bilateral ilio obturator lymphadenectomy using both classical and laparoscopic techniques. 

The study was conducted according to the guidelines of the Declaration of Helsinki, obtaining informed patient consent, and approved by the Ethical Committee of the Municipal Emergency Clinical Hospital of Timisoara (approval number 81/12.12.2022).

Primary processing of surgical biopsies

The tissue biopsies were fixed in 10% buffered formalin for 24-48 hours, after which they were paraffin-embedded following all the mandatory steps required to obtain paraffin blocks: formalin-fixed paraffin-embedded (FFPE). After an initial study of all FFPE specimens containing primary tumors and all lymph node groups obtained from bilateral ilio obturator lymphadenectomy, we selected for each case two blocks: one block of the primary tumor and one block of the lymph node, which we considered significant for our study. From each FFPE block, sections of 3µ were made and subsequently stained using the hematoxylin-eosin method. After dehydrating and mounting, the slides were scanned using the Ocus 20 scanner microscope (OCUS, Helsinki, Finland), followed by digital archiving and uploading in the Qu-Pass digital image analysis system (version 0.4.2.) for evaluation of the presence and types of TLSs in cervical cancer specimens related to the malignant tumor areas. The digital slides were archived in the Slide Library Center provided by 3DHistech (Budapest, Hungary).

The inclusion criteria for TLS evaluation and classification are as follows: (1) the microscopic identification of lymphoid structures organized as round or oval-shaped groups of lymphocytes around a blood vessel; (2) the presence or absence of germinal centers; (3) the presence of nerve components within TLSs; (4) TLS density and their topography related to tumor lesion; and (5) the presence or absence within TLS of metastatic malignant cell islets.

The exclusion criteria applied for selecting cases are as follows: (1) small samples that did not contain stromal components; (2) damaged samples resulting from an improper or prolonged formalin fixation; (3) microscopic sections with artifacts of cutting and staining; (4) patients who had an incomplete profile of parameters selected for the present study; and (5) patients who did not agree to have their tissues used for research purposes. 

Correlations with clinicopathologic parameters

Tertiary lymphoid structures are microscopic stromal tissue lymphoid aggregates responsible for local antitumor immunity and response to immune checkpoint inhibitors. Their presence may have an impact not only on the local inflammatory microenvironment but also on the general inflammatory state during malignant progression and on the lymphovascular invasion of cervical cancer. The impact of TLSs on clinicopathological parameters in cervical cancer is completely unknown. Thus, we selected the following parameters: age (to detect differences between premenopausal and postmenopausal appearances of TLSs); white cell count (WBCs); eosinophils lymphocytes; neutrophils and monocytes count; tumor grade (G); lymphovascular invasion; and tumor, nodule, and metastasis staging (TNM) classification.

Statistical analysis was performed by applying correlation tests. Jamovi software for macOS devices (The Jamovi Project (2023) version 2.3, Retrieved from https://www.jamovi.org) was used for statistical analysis. We considered a p-value of < 0.05 to be statistically significant and a strong correlation to be statistically significant when p < 0.001. The presence of TLS types (total and TLS associated with nerve fibers) was correlated with age (premenopausal versus menopausal status), lymphovascular invasion, lymphocytes, eosinophils, and neutrophil counts, but also with G. 

## Results

Types of TLSs encountered in cervical cancer

From a topographical point of view, we could classify TLSs into TLS with stromal distribution and intratumoral TLS. The analysis of TLS in the cases included in the study demonstrated a morphological heterogeneity of TLS depending on the progressive stages of cervical cancer.

**Figure 1:**
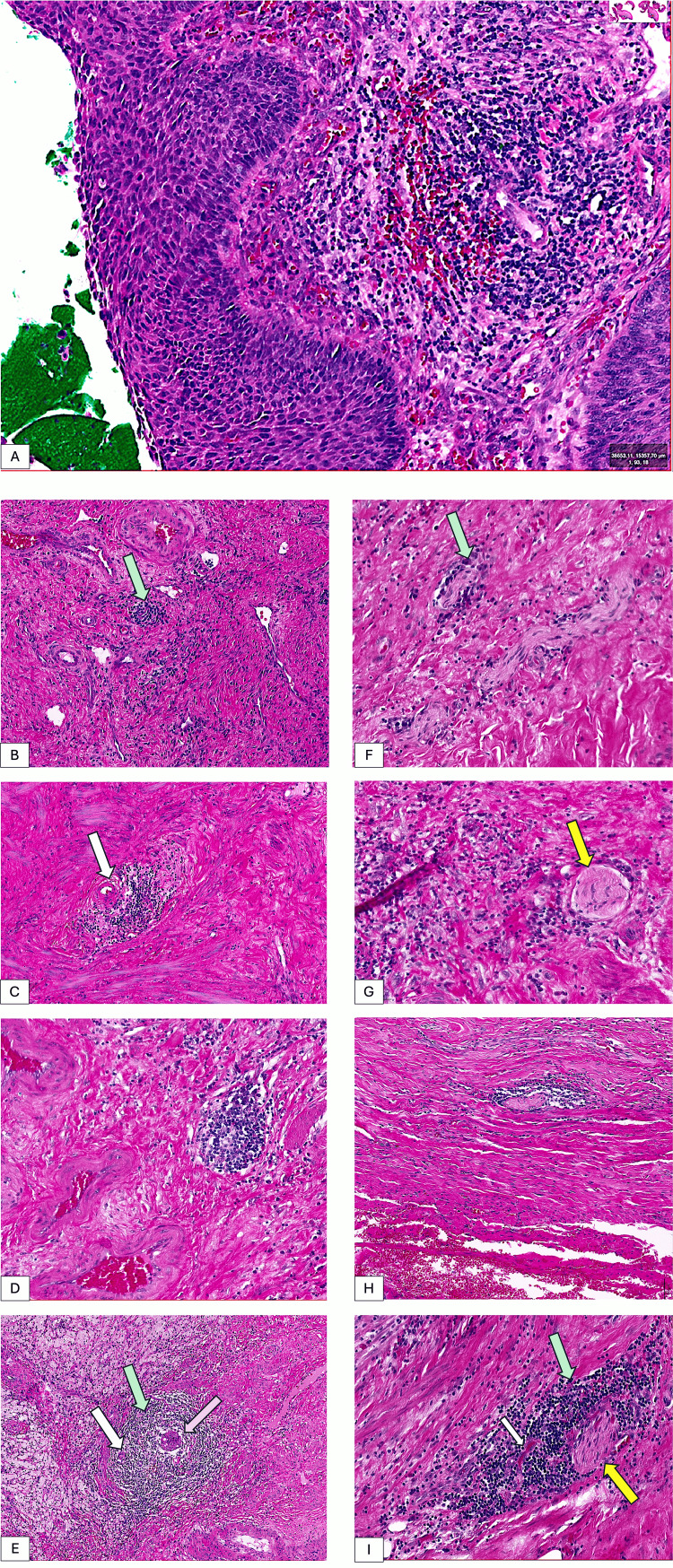
Stromal inflammation in cervical cancer appears early in its development from the pre-invasive phase (A). Stroma-associated inflammation is organized as diffuse inflammatory infiltrates (A) and two different types of TLSs. We detected the classic TLSs (B-E) composed of HEVs and lymphoid tissue and a particular form of TLSs where lymphoid tissue is associated with not only vascular components (HEVs, white arrows) but also nerve structures (named here TLSs.N, F-I). The developmental heterogeneity of both TLS types is noted. Classic TLSs contain lymphoid tissue (green arrow) and also vascular components, but they may contain metastatic foci (purple arrow), while TLSs.N are composed of lymphoid and vascular components but are always grouped around a nerve (yellow arrow). TLS: tertiary lymphoid structures; HEV: high endothelial venules; TLS.N: tertiary lymphoid structures associated with nerves

We first globally evaluated the total number of TLSs (TLS.T). At the level of the cervical stroma, we observed topographically two locations: TLSs that were immediately peritumorally positioned and TLSs that were distant from tumor lesions. The second category of TLS was found both in the surface epithelium, where the tumor could be detected and in the deep layers of the cervix. The development stages of TLS were also heterogeneous. In early lesions, the inflammatory infiltrate of the tumor stroma was diffuse type and restricted immediately below the non-keratinized squamous epithelium of the cervix. This aspect of diffuse but persistent inflammatory infiltration under the quasi-normal epithelium has been observed in the cervical areas adjacent to the tumor. Subepithelial inflammatory infiltrate from the early stages of cervical intraepithelial neoplasia (CIN) was accompanied by abundant vascularity at the borderline between diffuse inflammatory infiltrate and tumor (Figure 1A). The initial TLS in the early forms consisted of the existence of a small dilated vessel around which an organized accumulation of lymphoid tissue was observed (Figure 1B). It should be noted that, at this stage, the endothelial cells of the vessel that center the clumping of lymphoid tissue are flattened; they do not have the characteristics of high endothelial venules (HEVs). The size of these lymphovascular structures was small near the covering epithelium, but the density of the perivascular lymphocyte infiltration increased significantly once this type of stromal TLS was localized peritumorally.

Diffuse inflammatory infiltrate is also organized around malignant micro-invasion areas, together with adjacent small-sized TLS with high-visible endothelial vessels without germinal centers. This suggests an early activation of immunity in the process of stromal invasion. 

In invasive carcinomas, TLSs found superficially are larger and have a well-defined germinal center from a morphological point of view (Figures 1C, 1D). Tertiary lymphoid structures grow in size with the increase in proximity to the tumor, so we will find the largest germinal centers and venules with a highly visible endothelium (Figure 1D). In peritumoral TLS, we have seen in their composition tumor cell islets that seem to "metastasize" the TLS process that closely mimics lymph metastasis (Figure 1E). Among the peritumoral TLS, we observed the existence of structures like these that included (limiting) an area, an island of tumor cells. It should be noted that tumor cell islets were always completely surrounded by lymphoid tissue. We observed the structural heterogeneity of peritumoral TLS. In addition to the TLS described above with germinal centers and HEVs, it was observed in a proportion of 25% of TLS presence that had peripheral nerve structures embedded in lymphoid tissue, so they were named as TLS associated with peripheral nerves (TLS.N, Figures 1F-1I). The number of nerve structures encountered in a peritumoral TLS ranged from one to four, typically with peripheral distribution within the TLS.N. Of these cases, 42.85% had G3, while the remainder were classified as G2. Also, in all the cases we detected, TLS-N presented lymphovascular invasion. Additionally, in cases with TLS.N, we observed the existence of TLS associated with dilated lymphatic vessels. Intratumoral TLSs were always centered by a vessel with HEVs and rarely exhibited lymphoid follicles, and lymphocyte density was increased. Most intratumoral TLSs were observed in the periphery of tumor sites. It is worth mentioning the massive dilatation of the vessels inside the tumor and their irregular appearance, which suggests that the intratumor TLSs are most likely organized around not only pre-existing vessels but also the neo-formation vessels that invade the tumor. We can now describe two types of intratumoral TLSs in cervical cancer: peripherally distributed TLSs that include two or more tumor vessels that are most likely dilated, and centrally distributed TLSs with a structure similar to stromal TLS without lymphoid follicles. We did not detect TLS.N inside malignant areas.

In conclusion, our study highlights the topographical and morphological heterogeneity of TLSs in both the stromal compartment and the tumor compartment. Therefore, at the next stage of our study, we investigate whether this morphological and topographical heterogeneity has an impact on the clinical-pathological and biological parameters of the patients included in the study.

Impact of TLS on clinicopathological prognostic parameters

We have made correlations with age, the degree of tumor differentiation, and lymphovascular invasion. Furthermore, since TLSs are composed of inflammatory elements of the leukocyte type, we have made correlations between the microscopic aspects of TLS and the different categories of leukocytes, such as eosinophils, neutrophils, lymphocytes, and monocytes.

The TLS.T did not correlate statistically significantly with age; instead, we observed that 85.71% of the patients who presented TLS.N ages ranged between 59 and 72 years. For this, we obtained a statistically significant correlation (p = 0.005) between age and the presence of TLS associated with nervous structures. Also, a significant correlation between TLS.T and TLS.N was detected (p = 0.021%) (Table 1). 

**Table 1:** Correlation matrix between TLS type and age TLS: tertiary lymphoid structures; TLS.T: total number of TLS (with and without nerve structures inside); TLS.N: tertiary lymphoid structure associated with nerves

		TLS.N		TLS.T	Age
TLS.T	Pearson's r	0.378	*	—	
	df	27		—	
	p-value	0.021		—	
	Spearman's rho	0.378	*	—	
	df	27		—	
	p-value	0.021		—	
	Kendall's Tau B	0.378	*	—	
	p-value	0.023		—	
Age	Pearson's r	0.475	**	0.203	—
	df	27		27	—
	p-value	0.005		0.146	—
	Spearman's rho	0.468	**	0.228	—
	df	27		27	—
	p-value	0.005		0.118	—
	Kendall's Tau B	0.393	**	0.191	—
	p-value	0.007		0.114	—
Note: Hₐ is a positive correlation					
* p < .05, ** p < .01, *** p < .001, one-tailed					

A similar evolution of TLS parameters was seen for the degree of tumor differentiation. A statistically significant correlation between the degree of differentiation and TLS was only found for the TLS.N group, where the G3 degree of differentiation showed a p-value of 0.041 with TLS.N, and TLS.N did not statistically significantly correlate with G (Table 2).

**Table 2:** Correlation matrix between G and TLS TLS.T: total number of tertiary lymphoid structures; TLS.N: tertiary lymphoid structure associated with nerves; G: tumor grade

		TLS.N		TLS.T	G
TLS.T	Pearson's r	0.378	*	—	
	df	27		—	
	p-value	0.043		—	
	Spearman's rho	0.378	*	—	
	df	27		—	
	p-value	0.043		—	
	Kendall's Tau B	0.378	*	—	
	p-value	0.045		—	
G	Pearson's r	0.383	*	0.109	—
	df	27		27	—
	p-value	0.041		0.574	—
	Spearman's rho	0.383	*	0.109	—
	df	27		27	—
	p-value	0.041		0.574	—
	Kendall's Tau B	0.383	*	0.109	—
	p-value	0.043		0.565	—
Note: * p < .05, ** p < .01, *** p < .001					

Except in one case, patients who presented with TLS-N also experienced lymphovascular invasion and outbreaks of massive histiocytosis. For the first aspect, lymphovascular invasion resulted in a statistically significant correlation between TLS.N and lymphovascular invasion (p = 0.032, Table 3).

**Table 3:** Correlation matrix between TLS.N and lymphovascular invasion TLS.N: tertiary lymphoid structure associated with nerve fiber; LVI: lymphovascular invasion

		TLS.N		LVI
LVI	Pearson's r	0.348	*	—
	Df	27		—
	p-value	0.032		—
	95% CI Upper	1		—
	95% CI Lower	0.041		—
	Spearman's rho	0.348	*	—
	Df	27		—
	p-value	0.032		—
	Kendall's Tau B	0.348	*	—
	p-value	0.033		—
Note: Hₐ is a positive correlation				
Note: * p < .05, ** p < .01, *** p < .001, one-tailed				

The percentages of lymphocytes and monocytes in peripheral blood leukocytes correlated significantly with TLS.T included in the study. An inverse significant correlation with a p-value of 0.039 for lymphocyte count and a direct significant correlation with a p-value of 0.05 for monocyte count (Tables 4, 5) was found; but they did not correlate with TLS.N.

**Table 4:** Correlation matrix between TLS and lymphocyte count TLS.T: total number of tertiary lymphoid structures

		TLS.T		Lymphocyte %
Lymphocyte %	Pearson's r	0.332	*	—
	Df	27		—
	p-value	0.039		—
	Spearman's rho	0.294		—
	Df	27		—
	p-value	0.061		—
	Kendall's Tau B	0.244		—
	p-value	0.06		—
Note: Hₐ is a positive correlation				
Note: * p < .05, ** p < .01, *** p < .001, one-tailed				

**Table 5:** Correlation matrix between TLS.T and monocyte count TLS.T: total number of tertiary lymphoid structures

		Monocyte %		TLS.T
TLS.T	Pearson's r	-0.312	*	—
	Df	27		—
	p-value	0.05		—
	Spearman's rho	-0.276		—
	Df	27		—
	p-value	0.073		—
	Kendall's Tau B	-0.231		—
	p-value	0.072		—
Note: Hₐ is a negative correlation				
Note: * p < .05, ** p < .01, *** p < .001, one-tailed				

## Discussion

The tumor immune microenvironment is an important factor in the functioning of local and general immunity that the body develops as a defense and preservation reaction against tumor invasion. The normal appearance of the cervical stroma, which is made up of densely disordered connective tissue, involves a low density of lymphocytes or immune cells in the normal cervix. Inflammatory and hormonal lesions that develop in the cervix, especially in the exocol, cause the expansion of subepithelial capillary plexuses, followed by infiltration of the subepithelial area with inflammatory cells distributed diffusely throughout the affected epithelium [16,17].

Interest in the cervical immune microenvironment began to increase in the 2000s with the study of the effects of the human papillomavirus (HPV) and the development of the anti-HPV vaccine [18,19]. The link between the HPV virus and its carcinogenic capacity subsequently expanded the study of the immune microenvironment of HPV-induced cervical tumors. In addition to the heterogeneity of lymphocyte cells in tumor-associated inflammation and the heterogeneous organization of the cell types mentioned, a detailed analysis of stromal immune components has been shown. Organized immune structures in the form of lymphoid follicles have been observed in both the intratumor and the adjacent tumor stroma. These structures were classified as TLS and were considered an early local defense mechanism [4,11,12].

Tertiary lymphoid structures in cervical cancer have started to be studied intensively in the last two years, which has been closely related to the implementation of PDL-1 therapies, which are already applied in other types of malignancies but not in cervical cancer. These studies are, in fact, the most likely preliminary steps for initiating clinical trials of the use of immune-based therapies in cervical cancer. Data on morphology, heterogeneity, variability of cellular components, and the therapeutic and prognostic impact of TLS in cervical cancer are limited. At this point, only four articles referring to this issue are found in the literature [4,10,11,12]. Therefore, a series of extensive studies are needed to achieve a more complete, integrated clinical and therapeutic picture of the role of TLS in cervical cancer.

The structural, molecular, and topographical heterogeneity of TLS has been demonstrated in other types of malignancies, such as renal cancer [20], breast cancer [21], and oral cancer [22]. The majority of lesions in this study were squamous cell carcinomas. Heterogeneity of TLS has been observed in other types of squamous cell carcinomas, such as head and neck carcinomas [23], esophageal carcinomas [8], and squamous cell carcinomas starting in the lung [24].

For cervical cancer, there is no evidence of the heterogeneity of TLS, as well as the prognostic and therapeutic impact of these structures in the evolution of cervical cancer. In this study, we identified the heterogeneity of TLS in terms of morphology and topography. Based on the microscopic data, we found that the formation of TLS in cervical cancer begins early in the stages of cervical intraepithelial neoplasia, and their number and density increase with neoplastic progression. In invasive carcinomas, we identified TLS with both stromal and intradermal origins. Concerning intratumoral TLS, our data support two types of TLSs: multivascular TLSs distributed to the periphery of the tumor and containing two or more dilated vessels, and central TLSs with a structure similar to stromal TLS without lymphoid follicles. Most likely, the first type of TLS develops around neo-formation vessels, which has been described only in lung cancer to date [25].

The data described by Daum et al. indirectly refer to this by interrelating the application of antiangiogenic therapy with the impact of this therapy on TLS as a local antitumor defense mechanism. There are currently no similar data for other cancers, including cervical cancer. At the stromal level, we identified three types of TLS: no germinal center, germinal center, and associated peripheral nervous structures [25].

This study is the first to demonstrate the existence of a particular, rare form of TLS and TLS associated with peripheral nerves at the level of the cervical stroma. This type of TLS has also been described only in pancreatic cancer [26], where limited data in the literature suggest that it is associated with an improved survival rate in pancreatic cancer patients. In this study, the correlation between the presence of nerve-associated TLS and lymphovascular invasion suggests a negative prognostic factor in the progression and metastasis of cervical cancer. Data from our study also supported the predominance of TLS in people over 60 years of age, that is, in menopausal women. Another aspect that suggests that nerve-associated TLS may be a negative prognostic factor is the statistically significant association we found between the degree of G3 differentiation and peripheral nerve-associated TLS [26].

Also in pancreatic cancer, Zou et al. described the expression of the receptors for the estrogen receptors alpha and estrogen receptors beta in pancreatic adenocarcinoma as a positive prognostic factor, noting that, where estrogen receptors are expressed, infiltration with CD8+ T lymphocytes increases, as does the number of TLSs, suggesting the remodeling of the immune microenvironment as well as the simulation of the development of TLS. The structures of the cervix are also estrogen receptor-dependent tissues. Estrogen receptors are most likely to have a similar role in developing TLS in cervical cancer, but it is unclear whether this is achieved by the proliferative effect of estrogens on local lymphocytes or by increasing recruitment of peripheral lymphocytes that extravasate around stromal vessels with subsequent formation of TLS. Further extensive studies will be needed to prove this hypothesis [27].

As in other cancers and cervical cancers, the validation of classic biological markers is attempted and can be used as predictors of response to chemotherapy and radiotherapy. Related to the cellular component of TLS, a readily evaluable biomarker is represented by the percentage of peripheral blood lymphocytes and the existence of TLSs. Literature data describes the impact of this parameter or the neutrophil/lymphocyte ratio in peripheral blood on the postoperative prognosis of oncological patients, such as patients with squamous cell lung tumors [28] or urothelial carcinomas, where an increased number of TLSs associated with a reduced neutrophil/lymphocyte ratio has been reported to increase the response to pembrolizumab therapy [29]. In our study, the presence of an increased number of TLSs was significantly correlated with an increased number of peripheral lymphocytes.

Together with its strengths, the present study has several limitations. The main limitation of this study is due to the first description of TLS.N for cervical cancer. We have no other data to compare our results with the results from the other research centers involved in the study of cervical cancer stromal components as TLSs. The study was also limited because of its single-center design and the availability of data from patient records. Data collected from a single center may not be typical of the overall population, as patient background factors might vary significantly across the world.

Considering that the patient's addressability increased at our hospital for the diagnosis and treatment of cervical cancer, we will further conduct a comparative study on a larger number of patients in the next period. Tertiary lymphoid structures were significantly correlated to lymphovascular invasion and G3, and thus, we may assume that their presence may be considered a poor prognostic factor in cervical cancer. We will follow the evolution of the cases in this study for the disease-free period and determine if the cases in which we detected TLS.N. were relapses or distant metastases.

## Conclusions

We report here a particular form of TLS where a nerve component is associated with well-known components of classic TLSs. Tertiary lymphoid structures associated with nerves have not been previously described in cervical cancer, and no data about their impact on cervical cancer prognosis and aggressiveness are currently available. We found a significant correlation between TLS.N presence and age over 60, suggesting that TLS.N may be specific to menopausal women. They were also significantly correlated with lympho-vascular invasion and G3; therefore, we may assume that their presence may be considered a poor prognostic factor in cervical cancer. 
